# Playing RNase P Evolution: Swapping the RNA Catalyst for a Protein Reveals Functional Uniformity of Highly Divergent Enzyme Forms

**DOI:** 10.1371/journal.pgen.1004506

**Published:** 2014-08-07

**Authors:** Christoph Weber, Andreas Hartig, Roland K. Hartmann, Walter Rossmanith

**Affiliations:** 1 Center for Anatomy & Cell Biology, Medical University of Vienna, Vienna, Austria; 2 Max F. Perutz Laboratories, Department of Biochemistry and Cell Biology, University of Vienna, Vienna, Austria; 3 Institute of Pharmaceutical Chemistry, Philipps-University Marburg, Marburg, Germany; University of Michigan, United States of America

## Abstract

The RNase P family is a diverse group of endonucleases responsible for the removal of 5′ extensions from tRNA precursors. The diversity of enzyme forms finds its extremes in the eukaryal nucleus where RNA-based catalysis by complex ribonucleoproteins in some organisms contrasts with single-polypeptide enzymes in others. Such structural contrast suggests associated functional differences, and the complexity of the ribonucleoprotein was indeed proposed to broaden the enzyme's functionality beyond tRNA processing. To explore functional overlap and differences between most divergent forms of RNase P, we replaced the nuclear RNase P of *Saccharomyces cerevisiae*, a 10-subunit ribonucleoprotein, with *Arabidopsis thaliana* PRORP3, a single monomeric protein. Surprisingly, the RNase P-swapped yeast strains were viable, displayed essentially unimpaired growth under a wide variety of conditions, and, in a certain genetic background, their fitness even slightly exceeded that of the wild type. The molecular analysis of the RNase P-swapped strains showed a minor disturbance in tRNA metabolism, but did not point to any RNase P substrates or functions beyond that. Altogether, these results indicate the full functional exchangeability of the highly dissimilar enzymes. Our study thereby establishes the RNase P family, with its combination of structural diversity and functional uniformity, as an extreme case of convergent evolution. It moreover suggests that the apparently gratuitous complexity of some RNase P forms is the result of constructive neutral evolution rather than reflecting increased functional versatility.

## Author Summary

Many biocatalysts apparently evolved independently more than once, leading to structurally unrelated macromolecules catalyzing the same biochemical reaction. The RNase P enzyme family is an exceptional case of this phenomenon called convergent evolution. RNase P enzymes use not only unrelated, but chemically distinct macromolecules, either RNA or protein, to catalyze a specific step in the biogenesis of transfer RNAs, the ubiquitous adaptor molecules in protein synthesis. However, this fundamental difference in the identity of the actual catalyst, together with a broad variation in structural complexity of the diverse forms of RNase P, cast doubts on their functional equivalence. Here we compared two of the structurally most extreme variants of RNase P by replacing the yeast nuclear enzyme, a 10-subunit RNA-protein complex, with a single-protein from plants representing the apparently simplest form of RNase P. Surprisingly, the viability and fitness of these RNase P-swapped yeasts and their molecular analyses demonstrated the full functional exchangeability of the highly dissimilar enzymes. The RNase P family, with its combination of structural diversity and functional uniformity, thus not only truly represents an extraordinary case of convergent evolution, but also demonstrates that increased structural complexity does not necessarily entail broadened functionality, but may rather be the result of “neutral” evolutionary mechanisms.

## Introduction

RNase P is the endonuclease that generates the 5′ end of tRNAs by removing transcriptional extensions [1]–[3]. It is an indispensable enzyme found in essentially all forms of life. Despite the apparently simple function and the highly conserved structure of the tRNA substrates, a bewildering diversity of enzyme forms arose during evolution. Intriguingly, in many cases RNase P is a ribonucleoprotein (RNP), whose catalytic subunit is a structurally conserved RNA rather than a protein. In the most simple form of this RNP enzyme, found in Bacteria, the RNA is associated with a single small protein [4], [5], yet in Archaea, and even more so in the nucleus of Eukarya, the protein moiety of the RNP is complex, comprising up to 10 proteins (ranging in size from 15 to more than 100 kDa) [6]–[8]. While RNase P RNA is generally considered a relic of a primordial “RNA world” and thereby a kind of “living fossil” among modern-life's protein enzymes, it remains obscure why an RNA enzyme has been widely preserved for an endonucleolytic task that seems easily accomplishable by a protein. Likewise, the significance of the increasing complexity of the RNP's protein moiety during evolution is not understood, as the enzyme's tRNA substrates have remained essentially unchanged.

In contrast to the different RNPs, another major form of RNase P, only found in Eukarya, comprises a single monomeric 60-kDa protein (without any RNA subunit) that is called proteinaceous or protein-only RNase P (PRORP). This most simple, single-polypeptide-enzyme form acts in the nucleus and/or organelles of plants and some protists [9]–[11], as well as in animal mitochondria, where, however, two further protein subunits are involved in tRNA 5′-end maturation [12]. The identification of these RNase P forms exclusively composed of protein has revived the question as to why RNA catalysis has been retained by so many organisms just in the case of tRNA 5′-end maturation. It has also raised the issue whether RNase P forms structurally that different are at all functionally equivalent. In case of the nuclear RNase P forms found in different Eukarya the contrast in enzyme makeup is most striking: whereas a single protein apparently suffices in plants or trypanosomatids [10], [11], an RNP of considerable complexity evolved in the nucleus of animals and fungi [6]. This complexity has been suggested to increase the RNP enzyme's versatility, broadening its functionality, although the presumed substrates and functions beyond tRNA precursor processing remain poorly characterized [6],[7],[13]–[15]. Substrates apart from tRNA precursors have not been identified or studied so far for any of the nuclear protein-only forms of RNase P and it seems plausible that the full functional spectrum of the distinct RNase P forms may turn out to be quite diverse.

We recently addressed possible differences in the functional spectrum of RNA- and protein-based forms of RNase P by genetic complementation. In *Escherichia coli* we were able to rescue the otherwise lethal knockdown of the endogenous RNase P RNA by the expression of *PRORP1* from *Arabidopsis thaliana* [9]; the deletion of *Saccharomyces cerevisiae RPR1*, the gene encoding nuclear RNase P RNA, could be rescued by *Trypanosoma brucei PRORP1* [11]. While these experiments demonstrated a high degree of functional similarity/overlap between RNP and protein forms of RNase P, the consequences of the replacements were not further explored. Here we report the generation and characterization of yeast strains whose nuclear RNase P RNP was permanently replaced with the PRORP3 protein from *A. thaliana* by genome engineering. In addition to the molecular consequences of this swap at the level of RNA processing, we studied the strains under a variety of growth conditions to also address possible differences owing to hitherto unidentified functions of the RNP enzyme.

## Results

### PRORPs of Nuclear and Organellar Origin Are Able to Rescue the Deletion of Yeast Nuclear RNase P RNA

The RNA component and catalytic core of the yeast nuclear RNase P RNP is encoded by *RPR1* [6]. We recently showed that an otherwise lethal deletion of *RPR1* can be rescued by the expression of *T. brucei PRORP1*, the gene encoding the nuclear RNase P of this protist [11], from a plasmid. To test further *PRORP*s for their ability to substitute for yeast nuclear RNase P we set up a plasmid shuffle procedure (Figure S1). We were particularly curious to see whether PRORP variants naturally found in mitochondria or chloroplasts rather than the nucleus would be able to replace the nuclear RNP enzyme; accordingly, the organellar variants were expressed without their N-terminal targeting sequence.

The expression of any of the three *A. thaliana* or the two of *T. brucei PRORP*s allowed the tester strain to lose the *RPR1* expression plasmid harboring the *URA3* gene and thereby survive selection on 5-fluoroorotic acid (Table S1); only the human mitochondrial PRORP variant was not able to rescue the loss of *RPR1*. The ability to complement the deletion of *RPR1* depended on the enzymatic activity of the different PRORPs, as strains expressing active-site variants of the proteins were not able to survive. Loss of the *RPR1* expression plasmid and deletion of the chromosomal copy were verified by growth tests on selective media and PCR genotyping of the retrieved colonies (Figure S2). Although all PRORPs except for the human mitochondrial one supported the survival of the *RPR1* deletion strain after loss of the plasmid-borne *RPR1* copy, growth of the derived strains (as judged by colony size) differed markedly. Growth of strains based on *A. thaliana PRORP2* and *PRORP3*, or *T. brucei PRORP2* resembled that of the wild type; yeast cells expressing *T. brucei PRORP1* grew conspicuously slower and *A. thaliana PRORP1* supported growth most poorly. Consistent with a previously observed temperature sensitivity of *A. thaliana* PRORP2 [16], growth of the respective strain was impaired at 37°C. All the strains, even the slow-growing ones, could be maintained and re-grown in liquid or on solid media. Taken together, PRORPs of nuclear or organellar, protist or plant provenance provided sufficient (nuclear) RNase P activity for yeast cell survival.

### An “RNase P-Swapped” Yeast

**Figure 1:**
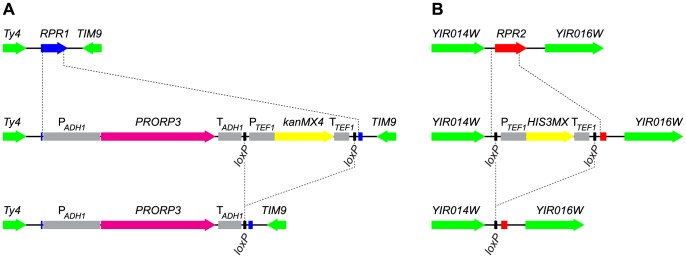
Gene replacement and deletion strategy: substitution of *RPR1* by *PRORP3* and deletion of *RPR2*. (A) The chromosomal *RPR1* gene was replaced with the *PRORP3* expression cassette (*A. thaliana PRORP3* driven by the *S. cerevisiae ADH1* promoter and terminator elements, and linked to a selectable marker (*kanMX4*)) by homologous recombination. (B) The chromosomal *RPR2* gene was replaced with the *HIS3MX* marker by homologous recombination. In both cases, the selectable markers (flanked by *loxP* sites) were subsequently removed by transient expression of Cre recombinase. *RPR1*-coding sequences are indicated in blue, *RPR2* in red, *PRORP3* in magenta, markers used for selection in yellow, flanking loci in green, relevant promoter and terminator elements in grey. Auxiliary lines indicate insertion/deletion points.

For an in-depth characterization of the consequences of the RNP-to-protein enzyme swap, we constructed strains in which *RPR1*, the gene encoding the catalytic RNA, was directly replaced by the gene expressing the protein catalyst. We opted for *A. thaliana PRORP3*, a nuclear RNase P in plants [10], which supported wild type-like growth in the plasmid-based complementation system. The genetic swap was achieved by homologous recombination of a *PRORP3* expression cassette (linked to a selectable marker) into the *RPR1* locus (Figure 1A). Haploid and diploid strains with a *PRORP3*-for-*RPR1* exchange (*rpr1Δ::PRORP3*) were viable, displayed wild type-like growth, and were able to mate and sporulate. Plant PRORP3 localized to the nucleus in yeast cells, showing a diffuse distribution without any apparent subnuclear (e.g., nucleolar) enrichment (Figure S3).

For the expression of *PRORP3* we used a truncated form of the *S. cerevisiae ADH1* promoter that was reported to be suitable for an efficient constitutive expression of heterologous proteins throughout the yeast growth cycle [17]. We also succeeded in the construction of haploid strains that expressed *PRORP3* from presumably weaker promoters, including the promoter of *POP1*, a gene encoding a subunit of the endogenous RNase P RNP. The growth of these strains (as judged by colony size), however, was substantially slower than that of strains with *ADH1* promoter-driven *PRORP3* expression, indicating that these promoters yielded only insufficient amounts of the alien enzyme. All further studies were thus confined to strains based on the *ADH1* promoter-*PRORP3* combination.

We aimed to also remove all further components of the former RNase P RNP presumably not needed anymore in an RNase P-swapped yeast. In addition to the catalytic RNA encoded by *RPR1*, the RNP comprises nine proteins, eight of which are, however, also integral components of another related and essential RNP, RNase MRP [6]. Rpr2p is the only protein specific to RNase P. Like *RPR1* and the other eight protein-coding genes, *RPR2* is normally essential for cell viability. We were nonetheless able to replace *RPR2* in *rpr1Δ::PRORP3* cells with a floxed marker (Figure 1B). Again, haploid and diploid *rpr1Δ::PRORP3 rpr2Δ0* strains were viable and their growth and physiology comparable to *rpr1Δ::PRORP3* or wild type cells.

To avoid subtle differences between the RNase P-swapped strains and the parental wild type strain, possibly resulting from unpredictable epistatic effects of auxotrophic markers or antibiotic resistance genes [18], we removed the markers used for selection during gene replacement (Figure 1). At all stages of strain construction, genotypes were verified by selective growth and PCR analyses (see Figure S4 for the analyses of the final diploid strains). At the final stage we isolated three clones each of the diploid *rpr1Δ::PRORP3*/*rpr1Δ::PRORP3* and *rpr1Δ::PRORP3rpr2Δ0*/*rpr1Δ::PRORP3 rpr2Δ0* strains and used them as replicates in all subsequent analyses.

### Levels of Mature tRNAs Are Unaffected by the RNase P Swap

**Figure 2:**
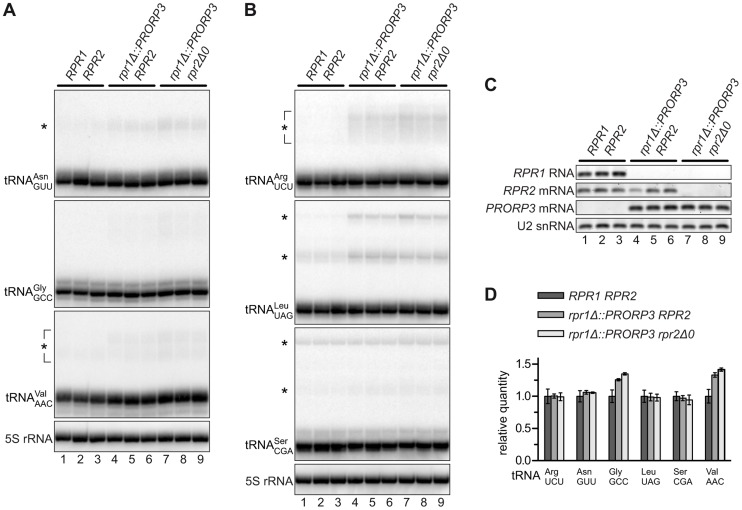
tRNAs in RNase P-swapped yeast strains. RNA was prepared from three independent clonal isolates of BY4743 and its RNase P-swapped derivatives, and analyzed by Northern blotting and RT-PCR. (A,B) Two blots were sequentially probed with oligonucleotides complementary to nucleus-encoded tRNAs and 5S rRNA. Blots were cropped to include the complete range of possible tRNA precursors (size estimates based on 5S rRNA hybridization signals). The relevant haploid genotypes of the homozygous diploid strains are indicated at the top. The RNA examined is specified to the left of each blot panel and presumed precursors indicated by asterisks. (C) The same samples were analyzed for the transcripts of the different RNase P genes by RT-PCR. (D) Quantitative analysis of 6 different tRNAs in RNase P-swapped yeast strains. Bands corresponding to mature tRNA were quantitated from the Northern blots (A,B) and normalized to 5S rRNA. Quantities are expressed relative to the mean of the parental BY4743 wild type strain. The mean and SD of the three clonal replicates are shown (see Table S2 for statistical analysis).

5′-end processing of tRNAs by RNase P is an early and essential step in tRNA biogenesis. We analyzed six nucleus-encoded tRNAs in the different RNase P-swapped yeast strains by Northern blotting. In all cases, hybridization signals corresponding to mature tRNA appeared indistinguishable between the parental wild type strain and both its RNase P-swapped derivatives (Figures 2A and 2B), although *RPR1* RNA (and *RPR2* mRNA) were, as expected, undetectable in the latter (Figure 2C). A quantitative analysis of the hybridization signals confirmed that the relative quantity of four of the six tRNAs was unaffected by the RNase P swap, and even slightly increased in the case of the remaining two (Figure 2D). In addition to the mature tRNA band, hybridization signals consistent with the size of tRNA precursors (and/or processing intermediates) were noticeable in the case of some of the analyzed tRNAs (Figures 2A and 2B). An increase of these precursor species in the PRORP3 strains was evident for tRNA^Leu^, while the putative tRNA^Ser^ precursor(s) remained unaffected by the RNase P swap. Diffuse hybridization signals in the precursor range were observed for tRNA^Arg^, tRNA^Asn^, and tRNA^Val^ in the PRORP3 strains, but not or only very weakly in the wild type cells. Essentially no precursors or processing intermediates could be detected for tRNA^Gly^, irrespective of the strains' RNase P status. Regardless of the precursor species identified, we like to emphasize that the mature tRNAs were qualitatively and quantitatively unimpaired by the RNase P swap in all analyzed cases.

### Putative Non-tRNA Substrates of Yeast Nuclear RNase P Do Not Accumulate in RNase P-Swapped Strains

**Figure 3:**
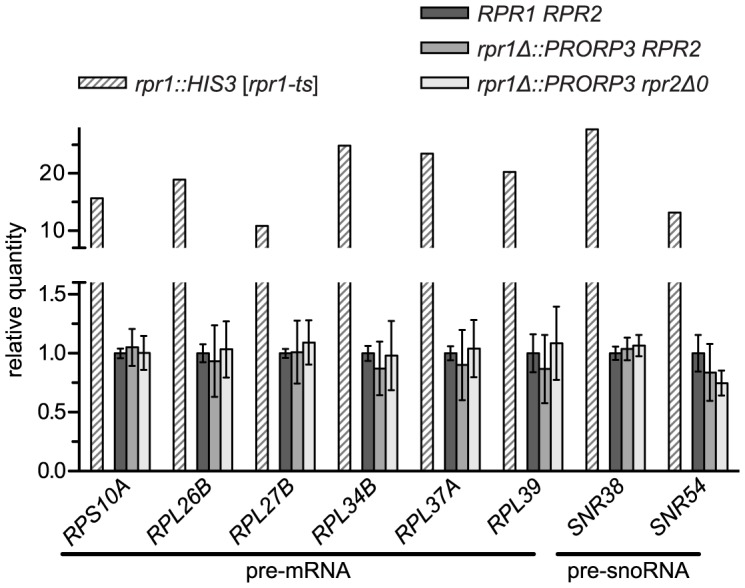
Levels of putative non-tRNA RNase P-substrates in RNase P-swapped yeast strains. RNA was prepared from three independent clonal isolates of BY4743 and its RNase P-swapped derivatives. Precursor RNAs that were previously reported to accumulate in a conditional RNase P-deficiency model [20], [21] were analyzed by quantitative RT-PCR. The RNase P deficiency strain JLY1 *rpr1::HIS3* [*rpr1-ts*] and its wild type counterpart JLY1 *rpr1::HIS3* [*RPR1*] were grown for two hours under restrictive conditions (37°C) and their RNA analyzed in parallel for comparison. Precursor RNA levels were normalized to the levels of *ACT1* and *CYC1* mRNA, and U6 snRNA. Quantities are expressed relative to (the mean of) the respective parental wild type strain(s) grown in parallel under identical conditions. The mean and SD of the three clonal replicates of the BY4743 wild type and of its RNase P-swapped derivatives, and the mean of technical duplicates of JLY1 *rpr1::HIS3* [*rpr1-ts*] are shown (see Table S2 for statistical analysis). Note that the y-axis is split into two segments of different scale to accommodate the entire range of variation.

Beyond tRNA precursors, yeast nuclear RNase P was proposed to be additionally involved in the processing of a broad range of non-tRNA substrates [6], [7], [13]–[15]. The RNP enzyme was reported to efficiently cleave RNA *in vitro* in an apparently structure- and sequence-independent way [19], and numerous unspliced mRNAs, snoRNA precursors, and other noncoding RNAs were found to either copurify with the enzyme or to accumulate in a temperature-sensitive model of RNase P deficiency [20], [21]. We selected eight of those most strongly accumulating RNAs and set up specific RT-PCR assays for their quantitative analysis. Using the temperature-sensitive *rpr1-ts* strain to validate our assay we could reproduce previous findings of a 10- to almost 30-fold accumulation of these mRNA and snoRNA precursors at the restrictive temperature. However, none of these RNAs was increased in either of the PRORP3-based strains (Figure 3).

The capability of the nuclear RNP enzyme to recognize and cleave such a diverse range of substrates was suggested to be related to the increased complexity of its protein moiety [6], [13]–[15]. Finding that the simplest form of RNase P, composed of a single polypeptide is apparently able to process the same wide range of substrates was surprising, and prompted us to evaluate the accumulation of the putative non-tRNA RNase P substrates in another RNase P-deficiency model. We made use of strains with titratable promoter alleles [22] to deplete yeast cells of RNase P proteins. The pertinent strain supposed to contain a repressible *RPR2* allele turned out to be flawed and we therefore used strains with repressible alleles of *POP4* and *POP8*, respectively. Depletion of either of the two proteins resulted in a marked decrease of *RPR1* RNA (Figure S5A), accumulation of tRNA precursors and reduced levels of mature tRNA (Figure S5B). The effect of the Pop4p depletion was more pronounced and comparable to that of the temperature-sensitive model after six hours, whereas 12 hours of depletion were required in the case of Pop8p to achieve similar effects. Remarkably, of the mRNA and snoRNA precursors strongly accumulating in the *rpr1-ts* mutant, none increased by more than a factor of 1.5, and several had even slightly reduced levels relative to the parental wild type strain (Figure S5C). In conclusion both, Pop4p and Pop8p depletion, proved to be appropriate RNase P-deficiency models, yet they did not recapitulate the accumulation of the supposed non-tRNA RNase P substrates, suggesting that the accumulation of those RNAs in the *rpr1-ts* mutant is not related to a deficiency in RNase P activity itself.

### RNase P-Swapped Yeasts Show Wild Type-Like Growth under a Wide Variety of Conditions

During strain construction and routine cultivation on solid or in liquid media the RNase P-swapped strains appeared largely indistinguishable from the wild type. Discerning more subtle differences in growth, however, requires quantitative analyses, and some functional deficiencies might only be revealed under specific conditions. Exploring a wide variety of growth conditions and different forms of stress moreover addresses cellular enzyme function in the broad range of its facets, including such of enzyme regulation and metabolic integration.

We used a high-throughput micro-scale cultivation approach with automated density reading to follow yeast growth throughout the complete cycle of adaption to the environmental change (lag phase), exponential growth, and stationary phase. From the growth curves we derived the maximal growth rate, the duration of the lag phase, and the final density reached at the endpoint (stationary phase). In pilot experiments, we determined the respective stressor concentrations that impaired, but did not prevent growth. Initial experiments involving both, *rpr1Δ::PRORP3* and *rpr1Δ::PRORP3 rpr2Δ0* strains, did not reveal any significant growth differences between the two RNase P-swapped strains. As the two genotypes had not differed in the RNA analyses too (see before), we restricted the comprehensive phenotypic analyses to a comparison of the *rpr1Δ::PRORP3 rpr2Δ0* genotype with the wild type.

**Figure 4:**
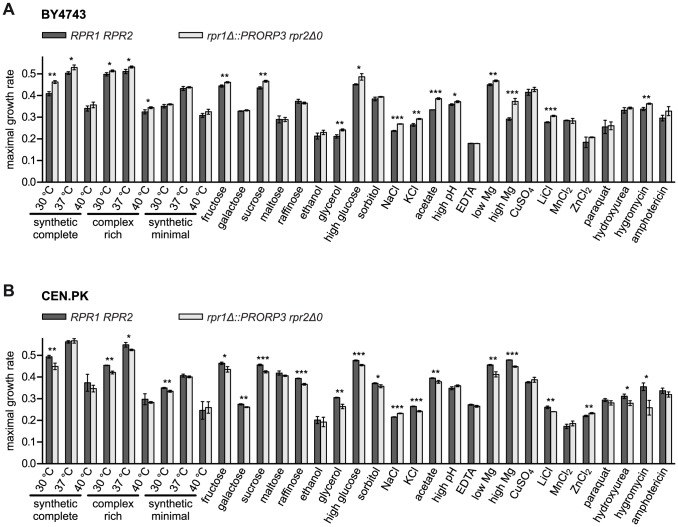
Quantitative phenotypic profiling of RNase P-swapped yeast strains. Three independent clonal isolates of BY4743 (A) and CEN.PK (B) wild type (*RPR1 RPR2*) strains and their respective RNase P-swapped (*rpr1Δ::PRORP3 rpr2Δ0*) derivatives were grown in suspension micro culture under different conditions. Media were inoculated with defined numbers of stationary starved cells and growth monitored by continual automated optical density reading until all cultures had apparently reached the stationary phase. Maximal growth rates were derived from the logarithmically transformed data. Growth was analyzed (i) in different standard media (synthetic complete, complex rich (YPD), or minimal (containing only essential components) medium with 2% glucose) at standard (30°C) or stress temperatures (37 and 40°C) each; (ii) in synthetic complete medium with different mono-, di-, or trisaccharides at 2% as fermentable carbon source, or with 3% ethanol or glycerol as a non-fermentable carbon source; (iii) under different forms of osmotic and/or ionic stress (16% “high” glucose, 1.5 M sorbitol, 1.0/0.5 M NaCl, 1.5 M KCl); (iv) under either organic acid (40 mM acetic acid) or alkaline stress (“high” pH; adjusted to 7.8 with Tris base); (v) under conditions of either limiting or increased availability of critical metal ions (330 µM EDTA (general depletion of trace metal ions), 42 µM “low” (100-fold lower than normal) MgCl_2_, 420 mM “high” (100-fold higher) MgCl_2_); and (vi) under different forms of toxic stress (333 µM CuSO_4_, 240/20 mM LiCl, 6 mM MnCl_2_, 8/4 mM ZnCl_2_, 600 µM paraquat, 100/20 mM hydroxyurea, 400/200 µM hygromycin B, 1.8/2 µM amphotericin) (in cases the conditions differed between the two strain backgrounds they are specified in the order BY4743/CEN.PK; unless otherwise specified, the basal medium was a synthetic complete medium containing 2% glucose). For a given condition all strains were analyzed in parallel. The mean and SD of the three clonal replicates are shown (*, P<0.05; **, P<0.01; ***, P<0.001; see Table S2 for P value listing); (A) BY4743-based strains; (B) CEN.PK-based strains.

Both types of RNase P, the native RNP as well as the transgenic protein, supported the growth of their respective strains under all the conditions tested (Figure 4). Surprisingly, a slight yet significant growth advantage was associated with the replacement of the RNP enzyme by PRORP3 for many of the conditions (reflected primarily in a higher maximal growth rate; Figure 4A). To assess the biological significance of this unexpected result, we decided to further examine whether genetic background affects the quantitative phenotype associated with the RNase P-swap. For comparison with the S288C background of the BY4743 parental strain [23] we chose CEN.PK, a distantly related, well-characterized laboratory strain with genomic relations to wild industrial strains [24], [25]. We reproduced the entire genetic exchange of RNase P in CEN.PK, again generating diploid *rpr1Δ::PRORP3* and *rpr1Δ::PRORP3 rpr2Δ0* strains. Like their BY4743-based cousins, the CEN.PK-based strains had normal levels of mature tRNA and did not accumulate any of the putative non-tRNA substrates of yeast nuclear RNase P (Figure S6).

The comparative phenotypic profile of the *rpr1Δ::PRORP3 rpr2Δ0* versus the wild type *RPR1 RPR2* genotype was qualitatively similar for the BY4743 and CEN.PK background, i.e., no condition exclusively allowed or strongly favored the growth of either one RNase P genotype (Figures 4, S7, and S8). However, in the BY4743 context, *PRORP3* under many growth conditions slightly raised the maximal growth rate of the cells (Figure 4A) and/or shortened their lag phase (Figure S7A), whereas the opposite was the case in the genetic context of CEN.PK (Figures 4B and S7B). The endpoint density reached was generally negatively correlated with these differences, i.e., faster growth resulted in a lower final density of the cultures (Figure S8). These basic effects of RNase P genotype and strain background were observed regardless of variations in media composition, type of carbon source, or nutrient availability, and no matter what kind of stress was applied. Only in the case of high salt (NaCl) stress the RNase P-swap resulted in a general growth advantage of PRORP3 strains (growth rate and endpoint) irrespective of the genetic background (Figures 4 and S8). However, like in essentially all other cases the difference was small; the only major change was a shortened lag phase for the CEN.PK RNase P-swapped strain in the presence of the fungicide amphotericin (Figure S7B). In summary, we did not find any serious growth deficiency when replacing the natural yeast RNase P RNP with *A. thaliana* PRORP3.

### The Long-Term Fitness Consequences of an RNase P Swap

**Figure 5:**
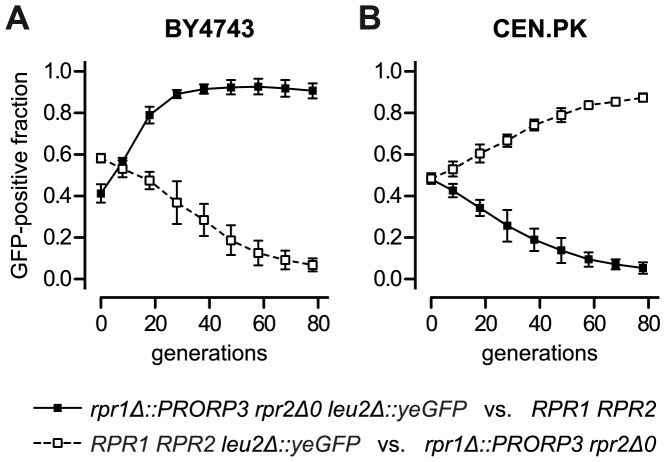
Competitive growth of yeast strains with ribonucleoprotein RNase P versus strains with protein-only RNase P. Wild type (*RPR1 RPR2*) BY4743 (A) and CEN.PK (B) were grown in co-culture with their respective diploid RNase P-swapped counterpart (*rpr1Δ::PRORP3 rpr2Δ0*) in glucose-containing synthetic complete medium. To distinguish the co-cultured strains they were mutually labeled with green fluorescent protein (GFP) by replacing one allele of the *leu2Δ0* and the *leu2-3_112* locus, respectively, with a *TEF1* promoter-driven, yeast-enhanced *GFP* (*yeGFP*-labeled wild type strains, open squares and broken line; *yeGFP*-labeled RNase P-swapped strains, filled squares and continuous line). Logarithmic starter cultures were mixed in equal proportion and a sample withdrawn for analysis. Co-cultures were grown to stationary phase, samples taken for analysis, and fresh medium was inoculated at 1:1000 with the stationary cells to reinitiate growth. Subsequently, seven full cycles of growth (each corresponding to ∼10 cell generations) and dilution were carried out with samples always taken at stationary phase. The fraction of GFP-positive cells in the samples was determined by flow cytometry. The mean and the 95% confidence interval of three experiments (from independent starting cultures) are shown; (A) BY4743-based strains; (B) CEN.PK-based strains.

As batch growth analyses generally involve only a limited number of generations (6–9 in our experiments), we devised a more long-term, competitive growth experiment to address possible fitness consequences of the RNase P swap. We labeled the *RPR1 RPR2* wild type and the *rpr1Δ::PRORP3 rpr2Δ0* strains by inserting an appropriate green fluorescent protein (*yeGFP*) expression cassette into one allele of their respective *leu2* locus. Pairs of wild type and RNase P-swapped strains, mutually labeled and unlabeled to exclude a possible GFP bias, were mixed in equal proportion and co-cultured. Rather than monitoring continuous (exponential) growth, we subjected the strain mixtures to repetitive cycles of batch culture, during each of which the cells traversed the phases of adaption, outgrowth, exponential growth, nutrient depletion, and stationary phase. Cells were sampled after each cycle and the fraction of GFP-positive cells was analyzed by flow cytometry. Consistent with the genetic background-dependent differences in growth rates and lag phases (Figures 4 and S7), the RNase P-swapped yeasts either outcompeted their parental wild type (BY4743 background; Figure 5A) or were themselves displaced (CEN.PK background; Figure 5B); after 78 generations the latest, all populations were homogenous to more than 90%. Hence, even without any evolutionary or experimental adaption, the artificial substitution of the endogenous RNP enzyme by a proteinaceous form of RNase P was, at least in a certain genetic background, able to produce strains that could outcompete the wild type.

## Discussion

Highly divergent forms of RNase P are found in the nucleus and organelles of different Eukarya. Besides their dissimilar composition and structure, these enzyme forms are also of distinct evolutionary origin. The RNA-based forms trace back to the origin of life, and, via a common ancestor with Archaea, developed to the complex RNPs found in the eukaryal nucleus; a bacterial-type RNase P was introduced with endosymbiotic organelles and either remained bacterial-like or evolved to the highly degenerate RNPs as found in extant mitochondria and plastids. Proteinaceous RNase P, in contrast, seems to have its origin (by fusion of functional domains) at the root of eukaryal evolution, possibly before the last eukaryal common ancestor. While its early evolution must have taken place in the presence of the RNP enzyme, the protein displaced the RNP in the nucleus and/or organelles of some eukaryal branches, and was lost in others. In contrast to the RNP forms, the protein-only enzyme apparently underwent little structural change and generally remained a “simple” 60-kDa monomer. The apparent discrepancy of convergent evolution towards tRNA processing function and divergent principles and trends in enzyme design has suggested that the complete functional spectrum of the disparate RNase P forms might actually differ noticeably. To put two of the structurally most divergent forms of RNase P to the litmus test for exchangeability and functional identity, we swapped one for the other in a suitable genetic model organism. We replaced the 10-subunit RNase P RNP of the yeast *S. cerevisiae* with *A. thaliana* PRORP3, a single plant protein. Finding that the artificial, RNase P-swapped strains were not only viable, but had essentially unchanged growth properties under a wide variety of conditions and, at least in a certain genetic background, a fitness that even slightly exceeded that of the wild type, demonstrated an unexpected extent of functional overlap, necessitating a revision of some of the current conceptions about RNase P function and evolution.

tRNA 5′-end maturation is the enzymatic function defining the RNase P family. The quantitative phenotypic and Northern blot analyses, indirectly and directly showed that there was no shortage of mature tRNAs in the RNase P-swapped yeasts; the alien plant enzyme was thus sufficiently expressed and active for a qualitatively and quantitatively proper maturation of tRNAs transcribed in the yeast nucleus. The major alteration done to the tRNA maturation machinery was nevertheless reflected in the appearance of precursors for some tRNAs. However, rather than indicating an intrinsic insufficiency of the plant enzyme, this more likely results from the change in the spatial organization of the natural pathway of yeast tRNA biogenesis in the RNase P-swapped strains. In line with this notion, the plant enzyme was distributed throughout the nucleoplasm rather than being confined to the nucleolus. This might actually reflect an inherent difference between early tRNA processing events in yeast and plants, nucleolar in the former [26] and nucleoplasmic in the latter [9]. Inappropriate localization, but also inadequate integration into the tRNA maturation pathway might moreover explain that a rather strong promoter was required to provide sufficient PRORP3, as it can reasonably be assumed that the plant protein lacks proper interactions with other components of the enzymatic machinery in yeast. Despite the unavoidable alterations of the natural pathway, the genetic RNase P swap demonstrates that PRORPs are fully proficient RNase P enzymes capable of faithfully processing an entire set of cellular tRNA precursors.

Apart from tRNA precursors, *E. coli* RNase P processes several other RNAs, the best characterized of which are 4.5S RNA and tmRNA [4]. Most of them mimic tRNAs with respect to the features critical for recognition by the bacterial enzyme. Similarly, tRNA-like structures in plant mitochondrial transcripts (t-elements) and in two human long noncoding RNAs were shown to be substrates for *A. thaliana* PRORP1 and human nuclear RNase P, respectively [9], [10], [27], [28]. In fact, any given RNase P's ability to cleave a certain RNA solely depends on the appropriate configuration of the structural determinants for interaction and cleavage site positioning, and not necessarily on a genuine tRNA structure. Thus, all types of RNase P are potentially involved in the processing of tRNA-related RNA structures, even though no such tRNA mimics have been identified in, e.g., yeast so far. The nuclear RNase P RNP, however, was proposed to have a substrate range that extends far beyond tRNA-resembling RNAs, and this versatility was related to the RNP's protein complexity [6], [7], [13]–[15]. The concept was based on the apparent propensity of the nuclear RNP enzyme to bind and cleave RNA in an apparently structure- and sequence-independent way [19], on the copurification of various RNAs with the enzyme [20], and, in particular, on the accumulation of a variety of different RNA (precursor) species in different models of conditional RNase P deficiency [20], [21], [29]. A selection of the RNAs most highly enriched in the best-characterized, temperature-sensitive *rpr1-ts* model failed to even slightly accumulate in our RNase P-swapped strains, strains whose phenotype and fitness did not point to any functional deficit in RNA processing either. The same RNA precursors also failed to accumulate in a novel model of RNase P deficiency when we tried to reproduce and verify the published findings, suggesting that their accumulation in the *rpr1-ts* mutant is an indirect, strain-specific effect and not directly caused by insufficient RNase P cleavage activity. It is worth noting that the rpr1-ts enzyme showed wild type-like activity at the restrictive temperature *in vitro* [30] and the molecular basis of the temperature-sensitive phenotype of the *rpr1-ts* strain thereby appears unclear. Furthermore, consistent *in vitro* cleavage by RNase P could not be demonstrated for any of the accumulating RNAs, and the RNA sets identified in the three previously published models (*rpr1-ts* and *pop1-ts* mutants; depletion of Rpp1p) were vastly different and showed an only negligible overlap [20], [21], [29]. This was attributed to either differences in the time course of the respective models, or the fact that all RNase P deficiency models except for *rpr1-ts* also affected the related RNP RNase MRP [20]. It is unclear, however, why presumptive RNase P substrates would not accumulate if RNase MRP were co-affected. In our RNase P-swapped strains RNase MRP was unaffected anyway and the largely unchanged phenotypic properties and fitness of the strains suggest that either the plant protein is able to process the same wide range of structurally diverse RNAs as the complex yeast RNP, or, more plausibly, the spectrum of RNA substrates of nuclear RNase P in yeast is much narrower than currently assumed, conceivably restricted to tRNA and tRNA-like structures.

Beyond RNA processing, still further functionality has been ascribed to the nuclear RNase P RNP. The human enzyme was reported to be required for transcription by RNA polymerase I and III through either direct transcriptional activation or chromatin remodeling; intriguingly, components of the RNP were reported to be recruited to chromatin stepwise, although the entire RNP holoenzyme was required for transcriptional activation [31], [32]. Based on a genetic interaction of *RPR1* with the RNA polymerase III transcription factor TFIIIB [33], a similar role in transcription was proposed for yeast nuclear RNase P [7], [34]. However, the straightforward replaceability of the yeast RNP by an unrelated plant protein appears not compatible with a crucial role of RNase P in transcription. Unless protein subunits of the RNP other than Rpr2p are responsible for transcriptional activation on their own, this would require *A. thaliana* PRORP3 to be productively engaged in the same interactions with chromatin and/or the transcriptional machinery as the yeast RNP. The latter possibility appears unlikely, and the lack of a phenotype that could have pointed to a specific functional deficit of the RNase P-swapped strains largely rules out any specialized function of the yeast RNP that cannot be accomplished by the protein enzyme. Surprisingly, the only phenotypic difference between wild type and RNase P-swapped strains consistently observed in both genetic backgrounds was a growth advantage of the latter under sodium chloride stress, which might hint that a monomeric protein is better able to cope with the associated changes of the intracellular milieu than the multi-subunit complex. The specific reasons for the otherwise mostly opposing growth and fitness differences of RNase P-swapped strains in the two genetic backgrounds remain unclear, either. The genomes of the *S. cerevisiae* strains BY4743 and CEN.PK are distinguished by a huge number of single nucleotide variations and small insertions/deletions, and by a few strain-specific genes [25], but none of those currently offers any obvious explanation for the genetic context-dependent fitness differences of RNase P-swapped strains.

Altogether our findings indicate that structural diversity and trends towards higher complexity within the RNase P family are not accompanied by functional diversification or increasing versatility. It seems that rather than broadening the enzymes functional spectrum as a result of positive selection, the increasing number of proteins in the nuclear RNP may have co-evolved with the RNA's loss of structural integrity through a process called constructive neutral evolution [35], [36]. According to this concept, the initially catalytically proficient RNase P RNA gathered RNA-binding proteins during evolution that fortuitously stabilized its structure. This stabilization allowed the RNA to accumulate destabilizing mutations, whereby its capacity to fold into the catalytically active conformation became dependent on the initially facultative binding partner(s). The dependency increased by a ratchet-like process, as further destabilization of the RNA structure was increasingly more likely than reversion to autonomy. Constructive neutral evolution was suggested to (at least in part) explain the evolution and gratuitous complexity found in some macromolecular machines, like, e.g., the spliceosome [35], [36]. The numerous proteins of the nuclear RNase P RNP would thus primarily serve to support and stabilize the RNA catalyst's structure. Consistently, even in the complex eukaryal RNP, the substrate specificity is primarily held by the RNA subunit, as the RNA alone is able to cleave tRNA precursors and model substrates at the correct cleavage site, although with very low efficiency [37]. The possibly only major functional diversification within the RNase P family, the RNase P/MRP split, contributes another relevant aspect. The two RNPs that obviously originate from a gene duplication of the ancestral RNase P RNA early in eukaryal evolution, (still) use essentially the same set of proteins to support their individual functions [3], [6]. If the protein subunits had a major role in conferring substrate specificity, the functions of the two RNP enzymes would be expected to either largely overlap, or the protein moieties would be expected to have diverged more markedly during eukaryal evolution. As the opposite of the two scenarios appears to be the case, the functions of RNase P and MRP must be essentially defined by their RNA moieties. Functionally distinct RNPs having a similar protein composition are actually not without precedent, and exemplified, e.g., by spliceosomal snRNPs and their common set of Sm proteins [38]. Intriguingly, the wide overlap in protein composition with RNase MRP is what might have rescued the nuclear RNase P RNP from extinction. Savings for a cell when substituting the functionally proficient, multi-subunit RNP with a single protein enzyme would be negligible, as (most) protein subunits would anyway have to be synthesized to maintain the essential functions of the sibling RNase MRP.

While it remains unclear why the RNA-based form of RNase P has been so widely preserved and a protein-only form exclusively “invented” in Eukarya, there are some further aspects of PRORP evolution that require mention. PRORP and RNP must have coexisted in the same genetic system, either nucleus or organelle, for quite some time in early Eukarya, yet there is currently no evidence for such redundancy in any living organism. This is surprising given that we did not observe a negative synthetic interaction of PRORP and the RNP enzyme during strain construction or plasmid shuffle. The ease with which the yeast nuclear system could be experimentally swapped from an RNP to a protein enzyme suggests that the evolutionary re-routing of PRORP to a different cellular compartment or its *de novo* acquisition from an endosymbiont, in contrast to that of a multi-component RNP, is a rather straightforward process. Whereas this might explain the apparent predominance of PRORPs in organellar systems [39], there is currently no solid evidence for the subcellular re-routing of an entire RNase P RNP. Our data also indicate that the common assertion that organellar RNase P enzymes are simpler than their nuclear counterparts, is probably incorrect. At least for PRORPs there seems to be no fundamental functional difference between nuclear and organellar forms; both were able to replace the yeast nuclear RNP enzyme. Taken together, the RNase P family seems not only characterized by a bewildering structural diversity, but also by a remarkably high degree of functional uniformity, and exemplifies convergent evolution at the enzyme level in an unprecedented extreme. While non-homologous, isofunctional (protein) enzymes are not an uncommon phenomenon as such [40], [41], and among the tRNA maturation enzymes exemplified by tRNA:m^1^G37 methyltransferases [42] or tRNA ligases [43], here an RNA and a protein independently evolved the same enzymatic activity and identical cellular function, a convergence of chemically unlike molecules apparently triggered by their substrates, tRNAs, a class of macromolecules that themselves have remained structurally unchanged throughout evolution.

## Materials and Methods

### Plasmids

The plasmids pFA6-kanMX4 [44], pUG6 [45], and pUG27 [46] were used as templates to prepare gene disruption/replacement cassettes by PCR. The *S. cerevisiae RPR1* gene, including ∼200 nucleotides of up- and downstream sequence, was PCR-cloned (for cloning primers see Table S3) into the *Eco*RI/*Pst*I sites of the 2μ plasmids YEplac195 (*URA3* marker) and YEplac181 (*LEU2*) [47]. The complete, uninterrupted coding sequences (without the organellar targeting sequence, where applicable) of the different *PRORP* genes were PCR-cloned into the *Xba*I/*Pst*I or *Xba*I/*Sca*I sites of a derivative of YEplac181 containing the truncated *S. cerevisiae ADH1* promoter that was generated as described previously [48]. Active-site mutants of *PRORP* genes were generated by site-directed mutagenesis using the QuikChange protocol (Agilent Technologies).

*A. thaliana PRORP3* together with the *ADH1* promoter was PCR-amplified from the *PRORP3*-YEplac181 construct and ligated together with a *S. cerevisiae ADH1*-terminator PCR fragment into the *Bsi*WI/*Sal*I sites of pUG6 [45]. pYM44 [49] was the template to prepare the *PRORP3*-*yeGFP*-tagging cassette. The *S. cerevisiae TEF1* promoter, the *yeGFP* coding sequence (from pYM44), and the *S. cerevisiae ADH1* terminator were PCR-amplified and ligated together into the *Bsi*WI/*Sal*I sites of pUG6.

CRE recombinase was expressed from pSH47 [45]. For all plasmids generated in the course of this study the inserted sequences were verified.

### Yeast Genetic Methods

Standard, complex rich (YPD), or glucose-containing synthetic complete, drop-out or minimal media were used, and standard techniques of cultivation and yeast genetics (selection on drop-out or antibiotic-containing plates, sporulation, tetrad dissection, mating, replica plating, etc.) employed [50], [51]; cells were grown at 30°C unless otherwise specified. For negative *URA3* selection (plasmid shuffle and removal of pSH47) appropriate dropout media were supplemented with 1 mg/ml 5-fluoroorotic acid and 50 µg/ml uracil. Plasmid DNA and PCR products were introduced into yeast cells by lithium acetate/polyethylene glycol-mediated transformation [52].

### Strain Construction

All *S. cerevisiae* strains generated in this study were derived from either BY4743 [23] or CEN.PK [24]. Gene disruption/replacement cassettes were prepared by PCR using primers with ∼40 nucleotides of homology to the target site at their 5′ end [53]. The primers with their targets and amplified genes/markers are listed in Table S4. The genotype of all new strains was confirmed by PCR using primers listed in Table S5.

One *RPR1* allele of BY4743 and CEN.PK, respectively, was replaced by the *A. thaliana PRORP3* expression cassette and *kanMX4* (Figure 1A), sporulation was induced and G418-resistant spores selected. Spores of opposing mating type were selected and mated after removing the floxed *kanMX4* [45] in one of them. In the case of the BY4743 background, spores of opposing genotype with respect to the heterozygous *LYS2* and *MET15* loci were selected, to recreate the *LYS2*/*lys2Δ0 MET15*/*met15Δ0* genotype of the parent strain. The remaining floxed *kanMX4*, and pSH47 were subsequently removed to create strains isogenic with their parents except for the *RPR1* locus. Three clones were selected from the final strain construction step. In the case of CEN.PK, *rpr1Δ::PRORP3*/*rpr1Δ::PRORP3* clones were obtained from different spores/mates and one of the wild type clones from a mate of two wild type spores (including transformation of plasmid based markers for selection of diploids and their subsequent removal). To delete *RPR2*, the gene was replaced with a floxed *HIS3MX* cassette [46] in two *rpr1Δ::PRORP3* spores of opposing mating type (Figure 1B). After the removal of *HIS3MX* in one of them, they were mated, and the remaining floxed *HIS3MX* and pSH47 removed from the diploids. Again, three clones, isogenic with their parent strains except for *RPR1* and *RPR2*, were selected from the final step.

For the plasmid shuffle experiments one allele of *RPR1* was disrupted in BY4743 using a *kanMX4* cassette (Figure S1). PRORP3 was GFP-tagged at its C-terminus in a BY4743 *rpr1Δ::PRORP3* strain using a *yeGFP*-*HIS3MX* cassette. Wild type BY4743 and CEN.PK, and their RNase P-swapped (*rpr1Δ::PRORP3 rpr2Δ0*) diploid derivatives were “GFP-labeled” by integration of a *yeGFP* expression cassette and *kanMX4* into one allele of the *leu2Δ0* and the *leu2-3_112* locus, respectively. The floxed *kanMX4* and pSH47 were subsequently removed.

The strain JLY1 (*rpr1::HIS3* [*RPR1*]) and its temperature-sensitive derivative (*rpr1::HIS3* [*rpr1-ts*]) were kindly provided by David R. Engelke [20], [21], [30]. Strain R1158 and the derivatives TH 5545 (P*_POP4_Δ*::tetO-P*_CYC1_*) and TH 3877 (P*_POP8_Δ*::tetO-P*_CYC1_*) were obtained from the yeast Tet-promoters Hughes collection (yTHC, Open Biosystems) [22]. *POP4* and *POP8* gene expression were shut down by addition of doxycycline to 50 µg/ml to the culture medium.

### RNA Analyses

Total cellular RNA was prepared by cell breakage with acid-washed glass-beads in a Precellys homogenizer and acidic guanidinium-phenol extraction using a commercially available reagent (RNAtidy G, AppliChem). 750 ng RNA were resolved by denaturing urea-polyacrylamide gel electrophoresis, electroblotted to nylon membrane, and cross-linked by UV. After prehybridization (6× SSC, 10× Denhardt's solution, 0.5% SDS, 100 µg/ml sheared, denatured salmon sperm DNA) the membranes were probed with ^32^P-labeled oligonucleotides in 6× SSC, 0.1% SDS over night at 42°C; oligonucleotide probes are listed in Table S6. Blots were sequentially washed with 6× SSC, 4× SSC, and 2× SSC, all with 0.1% SDS, subjected storage phosphor autoradiography, and quantitatively analyzed with ImageQuant TL 7 (GE Healthcare). Blots were stripped in 40 mM Tris·Cl (pH 7.6), 1% SDS, 0.1× SSC for 1 hour at 80°C before re-probing.

cDNA synthesis with gene specific primers and quantitative SYBR green real-time PCR was carried out as previously detailed [12]. The primers are listed in Table S7. Primary data analysis and normalization were carried out with the MxPro qPCR software (Agilent Technologies).

### Quantitative Phenotypic Profiling

The strains were grown to stationary phase in synthetic complete medium on 2% glycerol and 2% ethanol. Cells were washed twice with H_2_O, and suspended in sterile H_2_O at 5×10^6^/ml. Suspensions of these respiration adapted, stationary, starved cells were kept shaking at room temperature and used to start micro-cultures for phenotypic profiling. Starter-cultures prepared in this way gave reproducible results in growth-kinetic experiments over a period of at least 4 weeks.

Growth under different conditions was monitored in 96-well plates in 150 µl per well inoculated with 10^6^ cells. The plates were incubated at 30°C (unless otherwise indicated) in an EnSpire plate reader (PerkinElmer) with discontinuous “linear” shaking (60 sec at 1000 rpm, 0.5 mm amplitude; 36 sec pause) and automated turbidity readings at 600 nm taken every 10 min until all micro-cultures had apparently reached stationary phase. Growth data were processed in Excel (Microsoft) essentially as previously described [54], [55]. Optical densities were blank subtracted, corrected for non-linearity and path length, and finally log_e_-transformed. The maximal growth rate corresponds to the steepest slope that was stable (r^2^>0.995) for at least 4 hours and the lag phase to the time-intercept of the steepest slope [54]. Endpoints (final optical densities) were arbitrarily defined at the time when the growth rate had dropped below 0.025. Cultures in rich, complex medium did not reach a stationary phase during the experimental window; optical densities at the diauxic shift (sudden drop of the growth rate below 0.07) were derived instead.

### Competitive Growth Analysis

Starter cultures were grown in glucose-containing synthetic complete medium to early logarithmic phase and pairs of unlabeled and GFP-labeled strains mixed in equal proportion. A sample was withdrawn for analysis of the initial ratio and the co-cultures were grown to stationary phase. Samples were taken for analysis, and fresh medium inoculated at 1∶1000 to reinitiate growth. Subsequently, seven full cycles of growth and dilution were carried out with samples always taken at stationary phase. The samples were fixed with 4% paraformaldehyde and the fraction of GFP-positive cells in the samples was measured with an Accuri C6 flow cytometer (BD Biosciences).

### Statistical Analysis

The means of the three strains were compared by one-way ANOVA and, pairwise, by Tukey's multiple comparison test using Prism (GraphPad). The means of two strains were compared by unpaired Student's *t* test.

## Supporting Information

Figure S1 Plasmid shuffle procedure to test *PRORP* genes for their ability to rescue the deletion of *RPR1*. One copy of *RPR1* was replaced by a selectable marker (*kanMX4*) by homologous recombination (*rpr1Δ:: kanMX4*). After transformation of the cells with a plasmid-borne copy of *RPR1*, sporulation was induced and G418-resistant, uracil-prototrophic haploid cells were isolated. This strain was transformed with a plasmid-encoded *PRORP* expression cassette and the ability of *PRORP* genes to functionally replace *RPR1* tested by selection against the plasmid-borne uracil prototrophy (*URA3*) on 5-fluoroorotic acid (5-FOA).
(PDF)

Figure S2 Genotyping of RNase P-swapped yeast strains isolated by plasmid shuffle. DNA was prepared from colonies isolated after selection on 5-FOA and the deletion of *RPR1* verified by PCR of the deleted sequence and across the insertion sites of the marker gene. The analysis of a representative colony of each successful plasmid shuffle experiment with a different *PRORP* gene is shown (lane 2, positive control; see Table S1) and the genotype indicated at the top. The specific genotype examined is specified to the left of each agarose gel panel and the genotyping PCR indicated by a black bar above each gene cartoon to the right (blue, *RPR1*; yellow, *kanMX4*; grey, promoter and terminator of *kanMX4*; genes and PCR products drawn to scale).
(PDF)

Figure S3 Localization of *A. thaliana* PRORP3 expressed in *S. cerevisiae*. The coding sequence of a yeast-enhanced green fluorescent protein (yeGFP) was fused in-frame to the C-terminus of PRORP3 by integration at the 3′ end of the *rpr1Δ::PRORP3* locus. Yeast cells were fixed with paraformaldehyde, stained with Hoechst 33342 (DNA staining), and pictures taken by epifluorescence microscopy: (A) yeGFP-tagged PRORP3; (B) DNA staining; (C) bright-field view of the yeast cells with 5 µm scale bar.
(PDF)

Figure S4 Genotyping of the diploid RNase P-swapped yeast strains. DNA was isolated from three independent clonal isolates of BY4743 and its RNase P-swapped derivatives, and deletions and insertions were verified by PCR of deleted/inserted sequences and across insertion/deletion sites. The relevant haploid genotype of the homozygous diploid strains is indicated at the top. The specific genotype examined is specified to the left of each agarose gel panel and the genotyping PCR indicated by a black bar above each gene cartoon to the right (blue, *RPR1*; magenta, *PRORP3*; grey, promoter and terminator of *PRORP3*; red, *RPR2*; genes and PCR products drawn to scale).
(PDF)

Figure S5 *RPR1* RNA, (precursor) tRNAs, and presumptive non-tRNA RNase P substrates after Pop4p or Pop8p depletion. The R1158 P*_POP4_Δ*::tetO-P*_CYC1_* and R1158 P*_POP8_Δ*::tetO-P*_CYC1_* strains were grown (in parallel to the R1158 wild type control strain) in the presence of doxycycline to shut down the expression of *POP4* and *POP8*, respectively. RNA was prepared from cells harvested after 6 and 12 hours. (A) *RPR1* RNA was analyzed by quantitative RT-PCR and normalized to the levels of *ACT1* and *CYC1* mRNA, and U6 snRNA. The quantity is expressed relative to the wild type strain. The mean of technical duplicates is shown. (B) A Northern blot sequentially probed with oligonucleotides complementary to two nucleus-encoded tRNAs and 5S rRNA. The RNase P deficiency strain JLY1 *rpr1::HIS3* [*rpr1-ts*] and its wild type counterpart JLY1 *rpr1::HIS3* [*RPR1*] were grown for two hours under restrictive conditions (37°C) and their RNA analyzed in parallel for comparison (lanes 1 and 2). The relevant genotypes of the strains and the time of growth in the presence of doxycycline are indicated at the top. The RNA examined is specified to the left of each blot panel and presumed precursors indicated by asterisks. (C) Precursor RNAs that accumulate in the JLY1 RNase P-deficiency model (JLY1 *rpr1::HIS3* [*rpr1-ts*]) were analyzed after 6 hours of Pop4p and 12 hours of Pop8p depletion, respectively, by quantitative RT-PCR. Precursor RNA levels were normalized to the levels of *ACT1* and *CYC1* mRNA, and U6 snRNA. Quantities are expressed relative to the respective parental wild type strain grown in parallel under identical conditions. The mean of technical duplicates is shown. Note that the y-axis is split into two segments of different scale to accommodate the entire range of variation.
(PDF)

Figure S6 RNA analyses of the RNase P-swapped yeasts in CEN.PK strain background. RNA was prepared from three independent clonal isolates of CEN.PK and its RNase P-swapped derivatives, and analyzed by Northern blotting and (quantitative) RT-PCR. (A–C) Three blots were sequentially probed with oligonucleotides complementary to nucleus-encoded tRNAs and 5S rRNA. Blots were cropped to include the complete range of possible tRNA precursors (size estimates based on 5S rRNA hybridization signals). The relevant haploid genotypes of the homozygous diploid strains are indicated at the top. The RNA examined is specified to the left of each blot panel and presumed precursors indicated by asterisks. (D) The same samples were analyzed for the transcripts of the different RNase P genes by RT-PCR. (E) Quantitative analysis of 8 different tRNAs in RNase P-swapped yeast strains. Bands corresponding to the mature tRNA were quantitated from the Northern blots (A–C) and normalized to 5S rRNA. (F) Precursor RNAs that accumulate in the JLY1 RNase P-deficiency model (JLY1 *rpr1::HIS3* [*rpr1-ts*]; compare to Figure 3) were analyzed by quantitative RT-PCR. Precursor RNA levels were normalized to the levels of *ACT1* and *CYC1* mRNA, and U6 snRNA. (E,F) Quantities are expressed relative to the mean of the parental CEN.PK wild type strain. The mean and SD of the three clonal replicates are shown (see Table S2 for statistical analysis).
(PDF)

Figure S7 Quantitative phenotypic profiling of RNase P-swapped yeast strains (lag phase). Three independent clonal isolates of BY4743 (A) and CEN.PK (B) wild type (*RPR1 RPR2*) strains and their respective RNase P-swapped (*rpr1Δ::PRORP3 rpr2Δ0*) derivatives were grown in suspension micro culture under different conditions as described in the legend to Figure 4. Lag phases corresponding to the time-intercept of the steepest slope (maximal growth rate) were derived from the logarithmically transformed data. The mean and SD of the three clonal replicates are shown (*, P<0.05; **, P<0.01; ***, P<0.001; see Table S2 for P value listing); (A) BY4743-based strains; (B) CEN.PK-based strains.
(PDF)

Figure S8 Quantitative phenotypic profiling of RNase P-swapped yeast strains (endpoint density). Three independent clonal isolates of BY4743 (A) and CEN.PK (B) wild type (*RPR1 RPR2*) strains and their respective RNase P-swapped (*rpr1Δ::PRORP3 rpr2Δ0*) derivatives were grown in suspension micro culture under different conditions as described in the legend to Figure 4. Endpoint densities were arbitrarily defined as the optical densities (OD_600_) at the time when the growth rate had dropped below 0.025 (corresponds to a doubling time of more than 27.7 hours). For complex, rich medium the optical densities at the diauxic shift are given (sudden drop of the growth rate below 0.07) instead, as the cultures did not reach a stationary phase during the experimental window. Optical densities (OD_600_) were corrected for linearity and path length. The mean and SD of the three clonal replicates are shown (*, P<0.05; **, P<0.01; ***, P<0.001; see Table S2 for P value listing); in a few cases the cultures did not reach the endpoint during the observation period (n.d.). (A) BY4743-based strains; (B) CEN.PK-based strains.
(PDF)

Table S1 Ability of different *PRORP* genes to complement the deletion of *RPR1* as tested by plasmid shuffle.
(PDF)

Table S2 Statistical significances and P values of data from Figures 2D, 3, 4, S6E, S6F, S7, and S8.
(XLS)

Table S3 PCR primers used for plasmid cloning.
(PDF)

Table S4 PCR primers used to prepare the gene disruption/replacement cassettes.
(PDF)

Table S5 Primers used for genotyping PCRs.
(PDF)

Table S6 Oligonucleotide probes used in northern hybridizations.
(PDF)

Table S7 Primers used for RT-PCR.
(PDF)
