# Supplementary figures and images for: Playing RNase P Evolution: Swapping the RNA Catalyst for a Protein Reveals Functional Uniformity of Highly Divergent Enzyme Forms
Source: PLoS Genet. 2014 Aug 7;10(8):e1004506. doi: 10.1371/journal.pgen.1004506 (PMC4125048; doi:10.1371/journal.pgen.1004506)

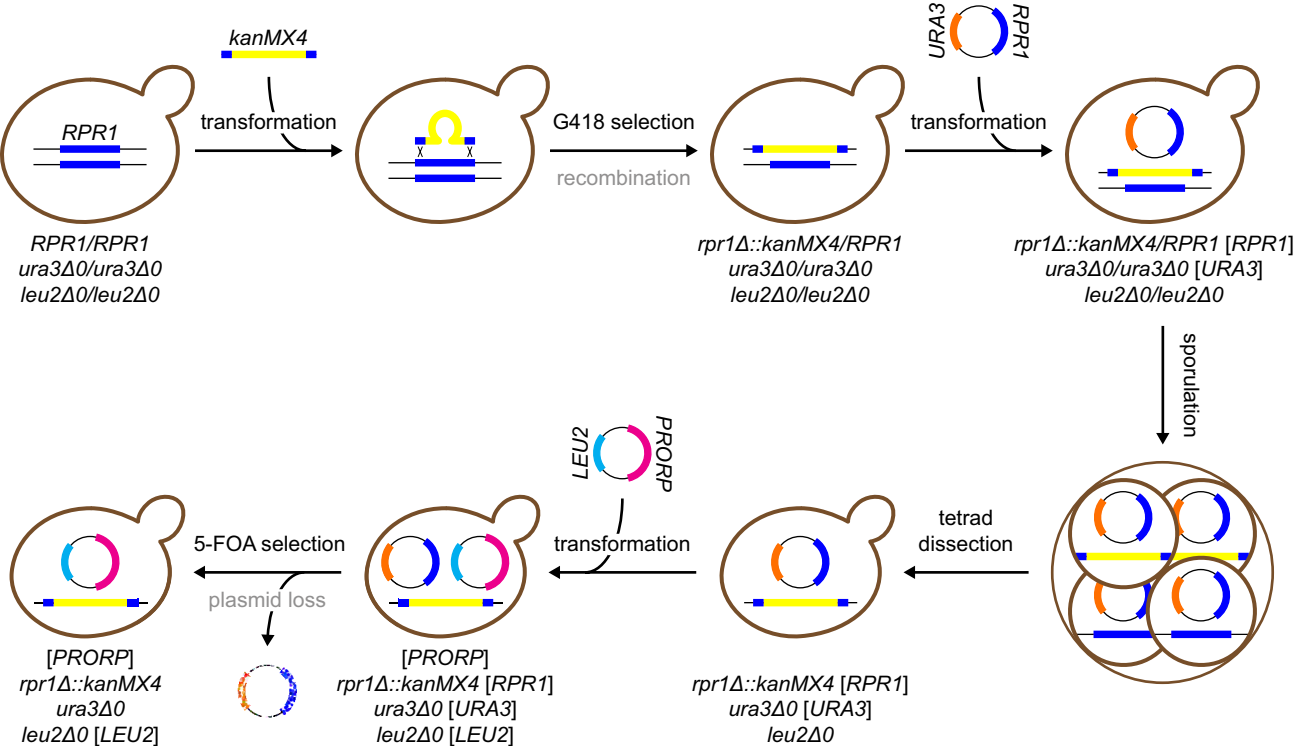

Supplement: Figure S1 — Plasmid shuffle procedure to test PRORP genes for their ability to rescue the deletion of RPR1. One copy of RPR1 was replaced by a selectable marker (kanMX4) by homologous recombination (rpr1Δ:: kanMX4). After transformation of the cells with a plasmid-borne copy of RPR1, sporulation was induced and G418-resistant, uracil-prototrophic haploid cells were isolated. This strain was transformed with a plasmid-encoded PRORP expression cassette and the ability of PRORP genes to functionally replace RPR1 tested by selection against the plasmid-borne uracil prototrophy (URA3) on 5-fluoroorotic acid (5-FOA). (PDF) [file pgen.1004506.s001.pdf]

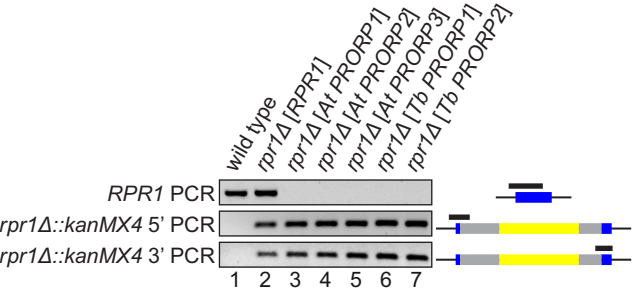

Supplement: Figure S2 — Genotyping of RNase P-swapped yeast strains isolated by plasmid shuffle. DNA was prepared from colonies isolated after selection on 5-FOA and the deletion of RPR1 verified by PCR of the deleted sequence and across the insertion sites of the marker gene. The analysis of a representative colony of each successful plasmid shuffle experiment with a different PRORP gene is shown (lane 2, positive control; see Table S1) and the genotype indicated at the top. The specific genotype examined is specified to the left of each agarose gel panel and the genotyping PCR indicated by a black bar above each gene cartoon to the right (blue, RPR1; yellow, kanMX4; grey, promoter and terminator of kanMX4; genes and PCR products drawn to scale). (PDF) [file pgen.1004506.s002.pdf]

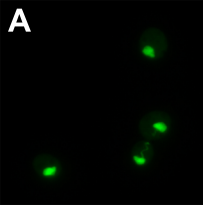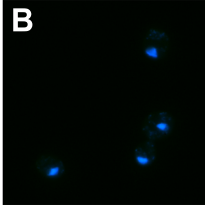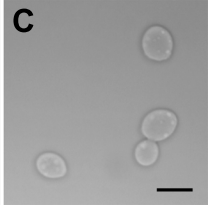

Supplement: Figure S3 — Localization of A. thaliana PRORP3 expressed in S. cerevisiae. The coding sequence of a yeast-enhanced green fluorescent protein (yeGFP) was fused in-frame to the C-terminus of PRORP3 by integration at the 3′ end of the rpr1Δ::PRORP3 locus. Yeast cells were fixed with paraformaldehyde, stained with Hoechst 33342 (DNA staining), and pictures taken by epifluorescence microscopy: (A) yeGFP-tagged PRORP3; (B) DNA staining; (C) bright-field view of the yeast cells with 5 µm scale bar. (PDF) [file pgen.1004506.s003.pdf]

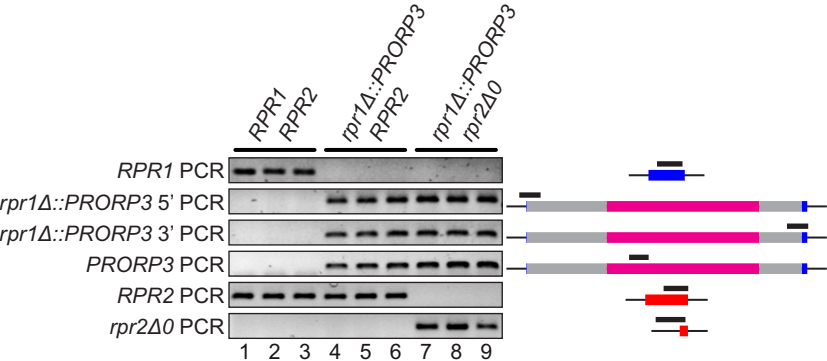

Supplement: Figure S4 — Genotyping of the diploid RNase P-swapped yeast strains. DNA was isolated from three independent clonal isolates of BY4743 and its RNase P-swapped derivatives, and deletions and insertions were verified by PCR of deleted/inserted sequences and across insertion/deletion sites. The relevant haploid genotype of the homozygous diploid strains is indicated at the top. The specific genotype examined is specified to the left of each agarose gel panel and the genotyping PCR indicated by a black bar above each gene cartoon to the right (blue, RPR1; magenta, PRORP3; grey, promoter and terminator of PRORP3; red, RPR2; genes and PCR products drawn to scale). (PDF) [file pgen.1004506.s004.pdf]

**A**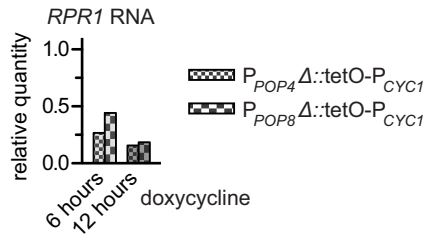**C**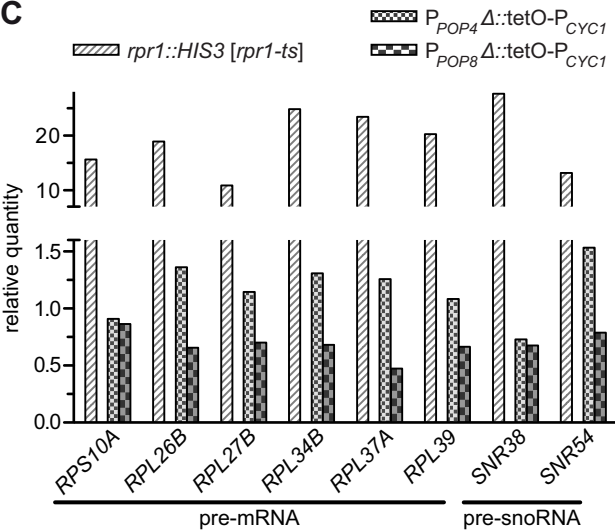**B**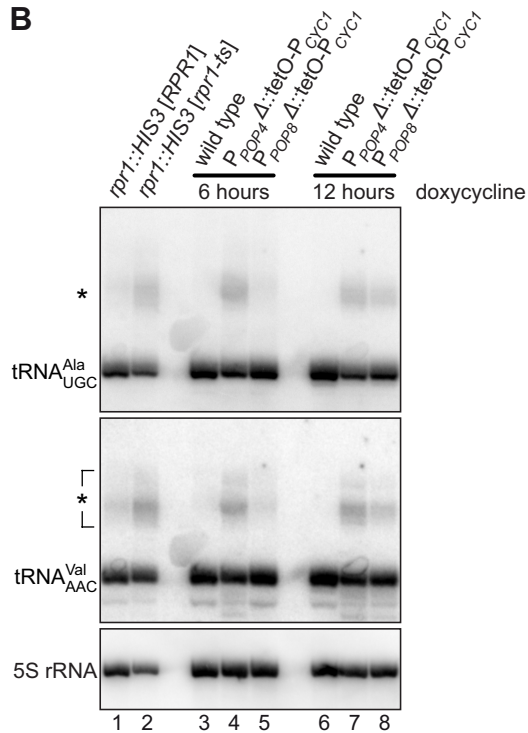

Supplement: Figure S5 — RPR1 RNA, (precursor) tRNAs, and presumptive non-tRNA RNase P substrates after Pop4p or Pop8p depletion. The R1158 PPOP4Δ::tetO-PCYC1 and R1158 PPOP8Δ::tetO-PCYC1 strains were grown (in parallel to the R1158 wild type control strain) in the presence of doxycycline to shut down the expression of POP4 and POP8, respectively. RNA was prepared from cells harvested after 6 and 12 hours. (A) RPR1 RNA was analyzed by quantitative RT-PCR and normalized to the levels of ACT1 and CYC1 mRNA, and U6 snRNA. The quantity is expressed relative to the wild type strain. The mean of technical duplicates is shown. (B) A Northern blot sequentially probed with oligonucleotides complementary to two nucleus-encoded tRNAs and 5S rRNA. The RNase P deficiency strain JLY1 rpr1::HIS3 [rpr1-ts] and its wild type counterpart JLY1 rpr1::HIS3 [RPR1] were grown for two hours under restrictive conditions (37°C) and their RNA analyzed in parallel for comparison (lanes 1 and 2). The relevant genotypes of the strains and the time of growth in the presence of doxycycline are indicated at the top. The RNA examined is specified to the left of each blot panel and presumed precursors indicated by asterisks. (C) Precursor RNAs that accumulate in the JLY1 RNase P-deficiency model (JLY1 rpr1::HIS3 [rpr1-ts]) were analyzed after 6 hours of Pop4p and 12 hours of Pop8p depletion, respectively, by quantitative RT-PCR. Precursor RNA levels were normalized to the levels of ACT1 and CYC1 mRNA, and U6 snRNA. Quantities are expressed relative to the respective parental wild type strain grown in parallel under identical conditions. The mean of technical duplicates is shown. Note that the y-axis is split into two segments of different scale to accommodate the entire range of variation. (PDF) [file pgen.1004506.s005.pdf]

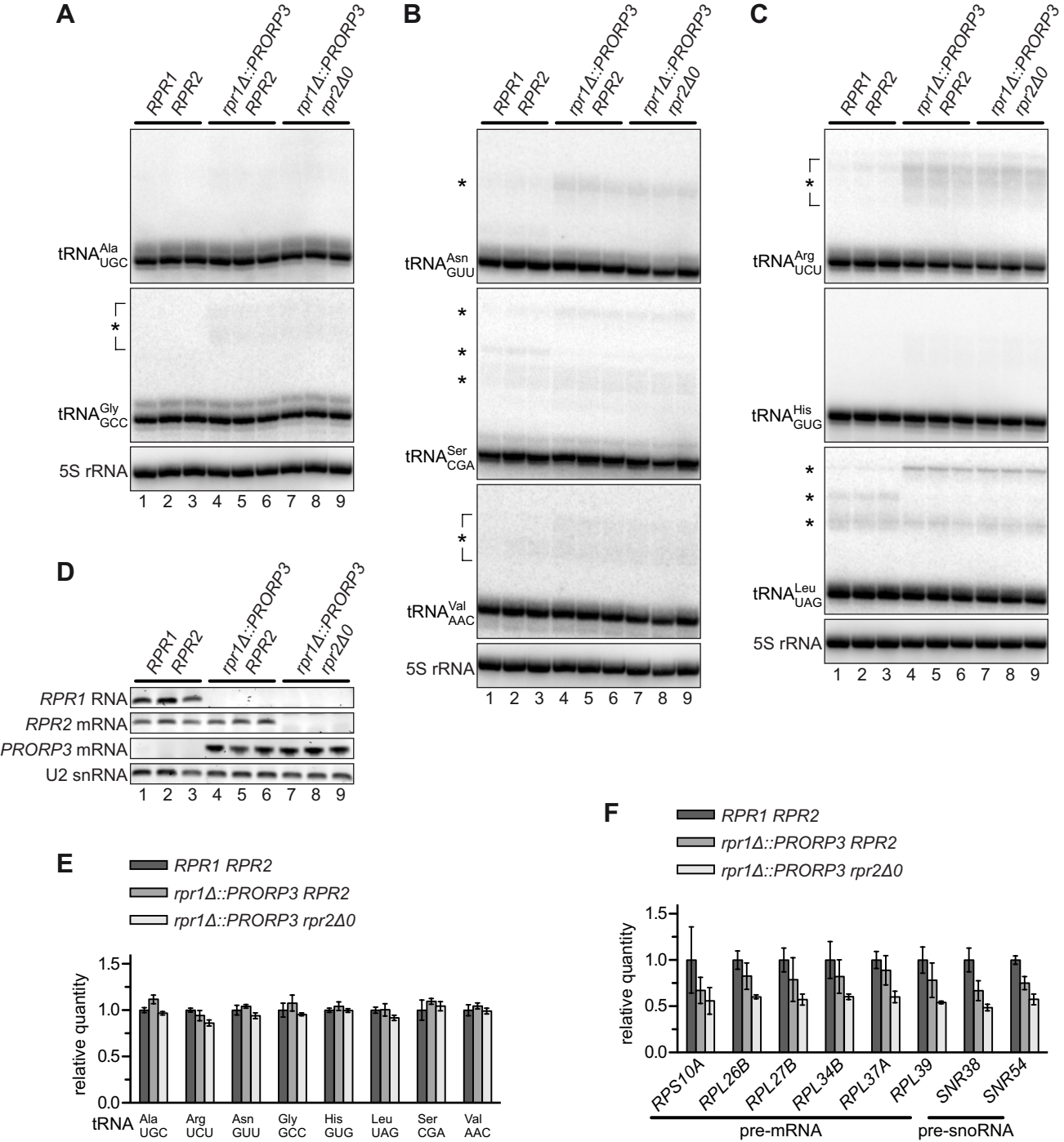

Supplement: Figure S6 — RNA analyses of the RNase P-swapped yeasts in CEN.PK strain background. RNA was prepared from three independent clonal isolates of CEN.PK and its RNase P-swapped derivatives, and analyzed by Northern blotting and (quantitative) RT-PCR. (A–C) Three blots were sequentially probed with oligonucleotides complementary to nucleus-encoded tRNAs and 5S rRNA. Blots were cropped to include the complete range of possible tRNA precursors (size estimates based on 5S rRNA hybridization signals). The relevant haploid genotypes of the homozygous diploid strains are indicated at the top. The RNA examined is specified to the left of each blot panel and presumed precursors indicated by asterisks. (D) The same samples were analyzed for the transcripts of the different RNase P genes by RT-PCR. (E) Quantitative analysis of 8 different tRNAs in RNase P-swapped yeast strains. Bands corresponding to the mature tRNA were quantitated from the Northern blots (A–C) and normalized to 5S rRNA. (F) Precursor RNAs that accumulate in the JLY1 RNase P-deficiency model (JLY1 rpr1::HIS3 [rpr1-ts]; compare to Figure 3) were analyzed by quantitative RT-PCR. Precursor RNA levels were normalized to the levels of ACT1 and CYC1 mRNA, and U6 snRNA. (E,F) Quantities are expressed relative to the mean of the parental CEN.PK wild type strain. The mean and SD of the three clonal replicates are shown (see Table S2 for statistical analysis). (PDF) [file pgen.1004506.s006.pdf]

**A****BY4743**■ *RPR1 RPR2*□ *rpr1Δ::PRORP3 rpr2Δ0*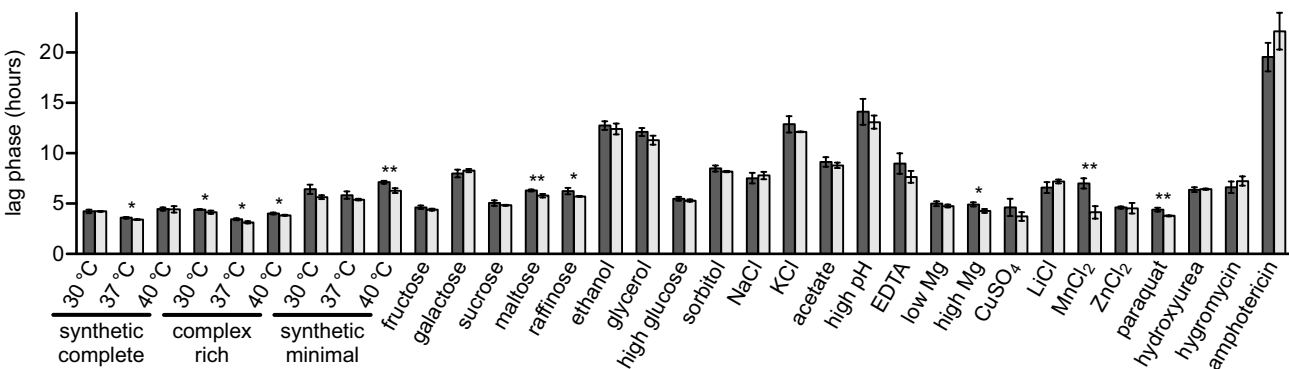**B****CEN.PK**■ *RPR1 RPR2*□ *rpr1Δ::PRORP3 rpr2Δ0*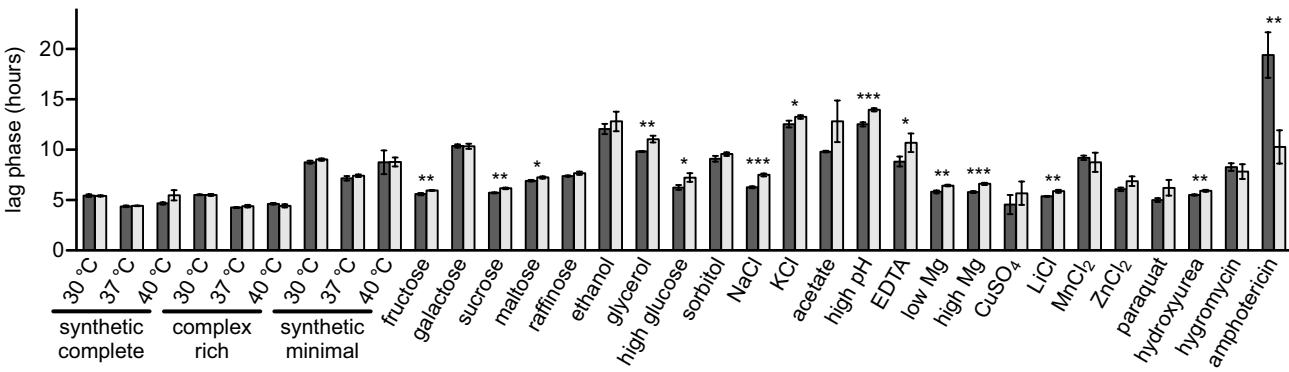

Supplement: Figure S7 — Quantitative phenotypic profiling of RNase P-swapped yeast strains (lag phase). Three independent clonal isolates of BY4743 (A) and CEN.PK (B) wild type (RPR1 RPR2) strains and their respective RNase P-swapped (rpr1Δ::PRORP3 rpr2Δ0) derivatives were grown in suspension micro culture under different conditions as described in the legend to Figure 4. Lag phases corresponding to the time-intercept of the steepest slope (maximal growth rate) were derived from the logarithmically transformed data. The mean and SD of the three clonal replicates are shown (*, P<0.05; **, P<0.01; ***, P<0.001; see Table S2 for P value listing); (A) BY4743-based strains; (B) CEN.PK-based strains. (PDF) [file pgen.1004506.s007.pdf]

**A****BY4743**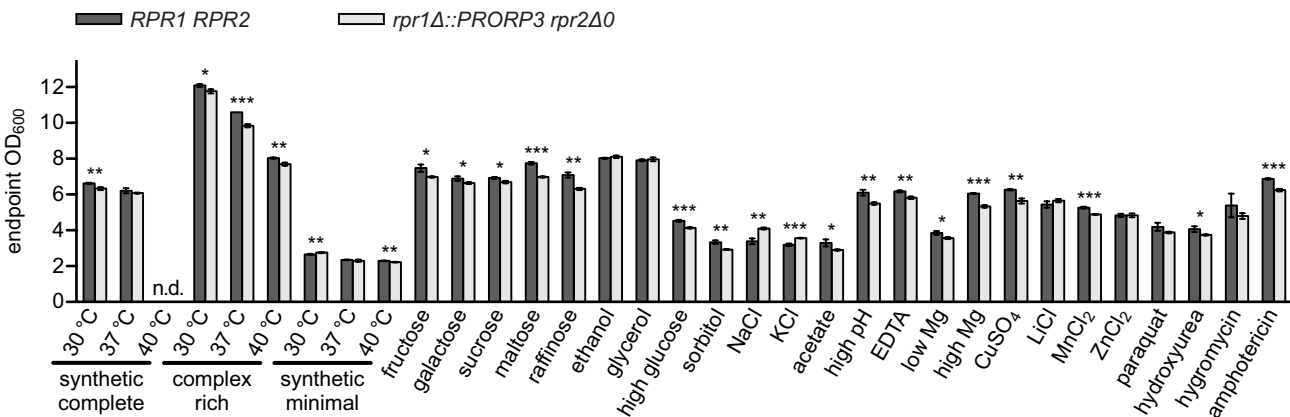**B****CEN.PK**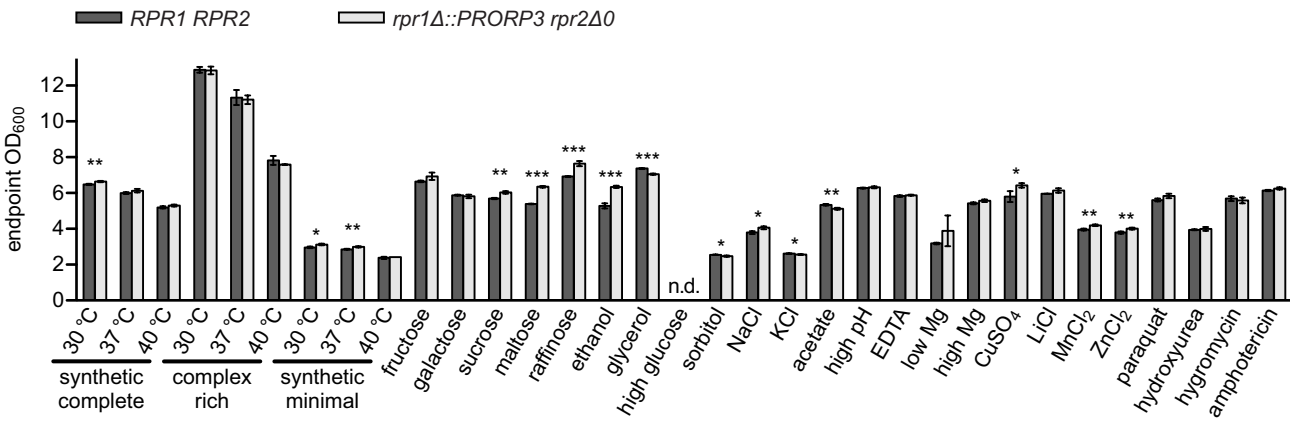

Supplement: Figure S8 — Quantitative phenotypic profiling of RNase P-swapped yeast strains (endpoint density). Three independent clonal isolates of BY4743 (A) and CEN.PK (B) wild type (RPR1 RPR2) strains and their respective RNase P-swapped (rpr1Δ::PRORP3 rpr2Δ0) derivatives were grown in suspension micro culture under different conditions as described in the legend to Figure 4. Endpoint densities were arbitrarily defined as the optical densities (OD600) at the time when the growth rate had dropped below 0.025 (corresponds to a doubling time of more than 27.7 hours). For complex, rich medium the optical densities at the diauxic shift are given (sudden drop of the growth rate below 0.07) instead, as the cultures did not reach a stationary phase during the experimental window. Optical densities (OD600) were corrected for linearity and path length. The mean and SD of the three clonal replicates are shown (*, P<0.05; **, P<0.01; ***, P<0.001; see Table S2 for P value listing); in a few cases the cultures did not reach the endpoint during the observation period (n.d.). (A) BY4743-based strains; (B) CEN.PK-based strains. (PDF) [file pgen.1004506.s008.pdf]
