# Supplementary material for: Playing RNase P Evolution: Swapping the RNA Catalyst for a Protein Reveals Functional Uniformity of Highly Divergent Enzyme Forms
Source: PLoS Genet. 2014 Aug 7;10(8):e1004506. doi: 10.1371/journal.pgen.1004506 (PMC4125048; doi:10.1371/journal.pgen.1004506)
Supplement: Table S1 — Ability of different PRORP genes to complement the deletion of RPR1 as tested by plasmid shuffle. (PDF) [file pgen.1004506.s009.pdf]

Table S1. Ability of different *PRORP* genes to complement the deletion of *RPR1* as tested by plasmid shuffle.

**RNase P gene**[a,b]	**Organism (natural subcellular localization)**	**Gene ID**	**Complementation**[c]
RPR1[d]	*S. cerevisiae* (nucleus)	9164884	yes
PRORP1	*A. thaliana* (mitochondria/chloroplasts)	817782	yes
PRORP1^{D475N}			no
PRORP2	*A. thaliana* (nucleus)	816166	yes
PRORP2^{D422N}			no
PRORP3	*A. thaliana* (nucleus)	828279	yes
PRORP3^{D422N}			no
PRORP1	*T. brucei* (nucleus)	3660621	yes
PRORP1^{D442N}			no
PRORP2	*T. brucei* (mitochondrion)	3664026	yes
PRORP2^{D417N}			no
PRORP	*Homo sapiens* (mitochondria)	9692	no

[a]*A. thaliana PRORP1*, *T. brucei PRORP2*, and human *PRORP* were expressed without their organellar targeting sequence.

[b]Superscript to gene names indicates the substitution of an invariant active-site aspartate residue presumably involved in the coordination of a catalytic metal ion, by asparagine. The substitution eliminates the cleavage activity of the recombinant protein.

[c]RNase P genes were tested for their ability to rescue the deletion of *RPR1* by plasmid shuffle as schematically described in Fig. S1: yes, colonies found on 5-FOA-containing medium; no, no colonies found on 5-FOA-containing medium; equal cell numbers were plated.

[d]Positive control: plasmid with leucine prototrophy (*LEU2*) carrying *RPR1* instead of a *PRORP* gene (Fig. S1).
