# Supplementary material for: Playing RNase P Evolution: Swapping the RNA Catalyst for a Protein Reveals Functional Uniformity of Highly Divergent Enzyme Forms
Source: PLoS Genet. 2014 Aug 7;10(8):e1004506. doi: 10.1371/journal.pgen.1004506 (PMC4125048; doi:10.1371/journal.pgen.1004506)
Supplement: Table S3 — PCR primers used for plasmid cloning. (PDF) [file pgen.1004506.s011.pdf]

Table S3. PCR primers used for plasmid cloning.

Gene[a]	Forward primer	Reverse primer	Cloning sites[b]
RPR1	GGCGAATTCACGACCCACATTGATAA	CAAGCTGCAGGCCGATAAGGTGTACT	*Eco*RI/*Pst*I
At PRORP1	CGTTCTAGACCATGGGCAGCCCATTTC	GCCTCTGCAGTCAAGGTGTTTTGGATCTT	*Xba*I/*Pst*I
At PRORP2	GCTCTAGACCATGGCTGCTTCTGAT	CGCCATGCATCTAAGGAATCTTCCCATTAC	*Xba*I/*Nsi*I
At PRORP3	GGCTCTAGACCATGGCTGGTACTGAT	CGCCATGCATCTATGAACTCTGCCTTGTA	*Xba*I/*Nsi*I
Tb PRORP1	CGTTCTAGACCATGGGTTCAGATT	GCCTATGCATCACCTATTTATGGCAG	*Xba*I/*Nsi*I
Tb PRORP2	CGTTCTAGACCATGGGGAGTATTCGACCT	CCCTATGCATCAGCGTACACATAA	*Xba*I/*Nsi*I
Hs PRORP	GGCTCTAGACCATGGGCTTTTCTC	GGGCATGCATAGCAGCCGGATCTCA[c]	*Xba*I/*Nsi*I
P_{ADH1}-*PRORP3*	GGACACGTACGGCCAGTGAATTCGAG[c]	AGCAGGTACCATGATTACGCCAAG[c]	*Bsi*WI/*Kpn*I
T_{ADH1}	GTGAGGTACCGGCGAATTTCTTATGA	GCAACTCGAGCTCGATTACAACAG	*Kpn*I/*Xho*I
P_{TEF1}	CGAGGCGTACGAATCCTTACATCACAC	GCGGATCCACCACACTTGTAAATC	*Bsi*WI/BamHI
yeGFP	CGGCTACAATTAATACATAACCT[c]	CCCGGTACCTTATTTAGAAGTGGC	BamHI/*Kpn*I

[a]Species origin of *PRORP* genes indicated as follows: *At*, *A. thaliana*; *Tb*, *T. brucei*; *Hs*, *H. sapiens*.
[b]Restriction sites used to prepare the PCR product for ligation; in some cases the sites were present in the PCR product rather than the primer.
[c]Primers to the plasmid from which the genes were amplified rather than to the gene sequence itself.
