# Supplementary material for: Playing RNase P Evolution: Swapping the RNA Catalyst for a Protein Reveals Functional Uniformity of Highly Divergent Enzyme Forms
Source: PLoS Genet. 2014 Aug 7;10(8):e1004506. doi: 10.1371/journal.pgen.1004506 (PMC4125048; doi:10.1371/journal.pgen.1004506)
Supplement: Table S4 — PCR primers used to prepare the gene disruption/replacement cassettes. (PDF) [file pgen.1004506.s012.pdf]

Table S4. PCR primers used to prepare the gene disruption/replacement cassettes.

Target gene	Forward primer	Reverse primer	Markers & genes[a]
RPR1	ACAGTGGTAATTCCTACGATTAAGAAACCTG TTTACAGAAGGCGCGCCAGATCTGTT	CAGACCTTGACGCTCACGCCGTAGCGGGCGA CAAGTCAAACTGGCGGCGTTAGTATCGAA	*kanMX4*
RPR1	CGGCTGGGAACGAAACTCTGGGAGCTGCGA TTGGCAGTGGAAGCTTCGTACGGCCAGTGA	GTAATCGGTATCGGGTTCGCCACTAATGACG TCCTACGATTGAGGCCACTAGTGGATCTG	*PRORP3* *kanMX4*
RPR2	GAACGGAAAAAAAAGATGAACAGAAAATAA AACAATACGTGGGGCCGCCAGCTGAA	GTAATTAGGATCGGCGCCAATATTAAAACGT TTAACGGTACCTATAGGGAGACCGGCAG	*HIS3MX*
rpr1Δ::PRORP3	AGGAATCTTTAAGAAGTTGGATGTGCATTAC AAGGCAGAGTTCACGTACGCTGCAGGTCG	GTAATCGGTATCGGGTTCGCCACTAATGACG TCCTACGATTGAGGCCACTAGTGGATCTG	*yeGFP* *HIS3MX*
LEU2	AGATCCATGTATAATCTTCATTATTACAGCCC TCTTGACCGCTACAATTAATACATAACC	TGTAGATTGCGTATATAGTTTCGTCTACCCTA TGAACATATTAGGCCACTAGTGGATCTG	*yeGFP* *kanMX4*

[a]Marker genes amplified and used to select for gene disruption/replacement and replacing genes, where applicable.
