# Supplementary material for: Playing RNase P Evolution: Swapping the RNA Catalyst for a Protein Reveals Functional Uniformity of Highly Divergent Enzyme Forms
Source: PLoS Genet. 2014 Aug 7;10(8):e1004506. doi: 10.1371/journal.pgen.1004506 (PMC4125048; doi:10.1371/journal.pgen.1004506)
Supplement: Table S5 — Primers used for genotyping PCRs. (PDF) [file pgen.1004506.s013.pdf]

Table S5. Primers used for genotyping PCRs.

Genotyping PCR[a]	Forward primer	Reverse primer
RPR1 PCR 1	CCTCGTGGCGCACATGGTA	GGCGACAAGTCAAACGGA
RPR1 PCR 2	GGAAATTCGGTGGAACACA	ATCGGGTTCGCCACTAATG
rpr1Δ::kanMX4 5’ PCR	CCTCGTGGCGCACATGGTA	GCGCACGTCAAGACTGTCAA
rpr1Δ::kanMX4 3’ PCR	GCGTCAATCGTATGTGAAT	TTGGATATGGGCTGGAAC
rpr1Δ::PRORP3 5’ PCR	CCTCGTGGCGCACATGGTA	GTATGGGTTTGGTTGCCAGA
rpr1Δ::PRORP3 3’ PCR	CTCCAGCAATGAGTTGATGA	TTGGATATGGGCTGGAAC
PRORP3 PCR	CGCGTTCATTAGTGACC	GACCGATACACCACCAA
RPR2 PCR	GCACGCAATTATATCAAATCAATG	CCCTAAGAATTTAGTAGATTACC
rpr2Δ0 PCR	CCTCTACTGAATATCGCCGAA	GCTCCGAGTAGGTCCTGTA

[a]PCRs specific for deleted/inserted gene sequences or across the insertion/deletion sites.
