# Supplementary material for: Playing RNase P Evolution: Swapping the RNA Catalyst for a Protein Reveals Functional Uniformity of Highly Divergent Enzyme Forms
Source: PLoS Genet. 2014 Aug 7;10(8):e1004506. doi: 10.1371/journal.pgen.1004506 (PMC4125048; doi:10.1371/journal.pgen.1004506)
Supplement: Table S6 — Oligonucleotide probes used in northern hybridizations. (PDF) [file pgen.1004506.s014.pdf]

Table S6. Oligonucleotide probes used in Northern hybridizations.

RNA specificity	Oligonucleotide sequence
$tRNA^{Ala}_{UGC}$	CCGATGACCTCTTCCTTGC
$tRNA^{Arg}_{UCU}$	CACTCACGATGGGGGTC
$tRNA^{Asn}_{GUU}$	CTCACGATCTTGCGATTAACAG
$tRNA^{Gly}_{GCC}$	GCAAGCCCGGAATCGAAC
$tRNA^{His}_{GUG}$	TGCCATCTCCTAGAATCGAAC
$tRNA^{Leu}_{UAG}$	CCCTTGCATCCGAAGATATCAGA
$tRNA^{Ser}_{CGA}$	CGACACCAGCAGGATTTGA
$tRNA^{Val}_{AAC}$	GGACGTTCTGCGTGTTAAG
5S rRNA	TGGTAGATATGGCCGCAAC
