# Supplementary material for: Playing RNase P Evolution: Swapping the RNA Catalyst for a Protein Reveals Functional Uniformity of Highly Divergent Enzyme Forms
Source: PLoS Genet. 2014 Aug 7;10(8):e1004506. doi: 10.1371/journal.pgen.1004506 (PMC4125048; doi:10.1371/journal.pgen.1004506)
Supplement: Table S7 — Primers used for RT-PCR. (PDF) [file pgen.1004506.s015.pdf]

Table S7. Primers used for RT-PCR.

Gene	Forward primer	Reverse primer
RPR1	GGAAATTCGGTGGAACACA	GGCGACAAGTCAAACGGA
RPR2	GCACGCAATTATATCAAATCAATG	GCTCCGAGTAGGTCCTGTA
PRORP3	CGCGTTCATTAGTGACC	GACCGATACACCACCAA
LSR1	GCTTGCTTATCCCCAAGTATC	CCATTTATATCCTGCGAGAAGAG
RPS10A	GTACGAACTAAGTAGCCAA	CCGTGTATCTTCATGCTTT
RPL26B	GTATCAAATATGGCTAAGCAA	GCTTAGGTACATTCCTCCTCATC
RPL27B	CGAAATCAATATAACCATGGCT	ATTTCCCATCTTATGCACCT
RPL34B	GATTAGCAACGAACAACCCT	TACGCTTCTCTAAATAGCACT
RPL37A	GCCTCATACAAATAATATAGACA	TAAGTATAAATTTGGTGTGCAT
RPL39	CAGATAGATCAACATGGCT	TTGTAGGTGGTAAGGTCAT
SNR38	TACATTGTTTTGCGCGTTT	GTTACCCATTCAGACAGGGAT
SNR54	TCTTTCATTAGAATGCGCTT	GATCACAGTCAGTAGAACGAA
ACT1	CGGTAGACCAAGACACCA	ATCTTTTCCATATCGTCCCA
CYC1	GATGTCTACAATGCCACAC	CTGACATGTTATTTTCGTCC
SNR6	GTAACCCTTCGTGGACATT	ATCTCTTTGTAAAACGGTTCAT
